# Taqman Real-Time PCR Detects *Avipoxvirus* DNA in Blood of Hawaìi `Amakihi (*Hemignathus virens*)

**DOI:** 10.1371/journal.pone.0010745

**Published:** 2010-05-27

**Authors:** Margaret E. M. Farias, Dennis A. LaPointe, Carter T. Atkinson, Christopher Czerwonka, Rajesh Shrestha, Susan I. Jarvi

**Affiliations:** 1 Department of Pharmaceutical Sciences, College of Pharmacy, University of Hawaìi at Hilo, Hilo, Hawaìi, United States of America; 2 Pacific Island Ecosystems Research Center, United States Geological Survey, Hawaìi National Park, Hawaìi, United States of America; Institute of Evolutionary Biology (CSIC-UPF), Spain

## Abstract

**Background:**

*Avipoxvirus* sp. is a significant threat to endemic bird populations on several groups of islands worldwide, including Hawaìi, the Galapagos Islands, and the Canary Islands. Accurate identification and genotyping of *Avipoxvirus* is critical to the study of this disease and how it interacts with other pathogens, but currently available methods rely on invasive sampling of pox-like lesions and may be especially harmful in smaller birds.

**Methodology/Principal Findings:**

Here, we present a nested TaqMan Real-Time PCR for the detection of the *Avipoxvirus* 4b core protein gene in archived blood samples from Hawaiian birds. The method was successful in amplifying *Avipoxvirus* DNA from packed blood cells of one of seven Hawaiian honeycreepers with confirmed *Avipoxvirus* infections and 13 of 28 Hawaìi `amakihi (*Hemignathus virens*) with suspected *Avipoxvirus* infections based on the presence of pox-like lesions. Mixed genotype infections have not previously been documented in Hawaìi but were observed in two individuals in this study.

**Conclusions/Significance:**

We anticipate that this method will be applicable to other closely related strains of *Avipoxvirus* and will become an important and useful tool in global studies of the epidemiology of *Avipoxvirus*.

## Introduction

Avian pox virus (*Avipoxvirus* sp.) has caused extensive morbidity and mortality in the native Hawaiian avifauna [1], [2] and currently threatens endemic birds in the Galapagos and Canary Islands [3]–[6]. The virus is mechanically transmitted on the mouthparts of blood or tissue feeding arthropods or by entry through cuts or breaks in the skin. Two types of disease have been described – localized cutaneous lesions at the site of viral entry and a disseminated diphtheritic form of infection where the virus spreads on mucous membranes of the mouth, esophagus, and upper digestive tract. Lesions can lead to blindness when they occur around the eyes, can obstruct feeding or breathing when they occur around the mouth or in the esophagus, can interfere with perching when they occur on the feet or legs, and frequently lead to development of secondary bacterial infections [7]. Early Hawaiian bird extinctions of the mid to late 1800's have been attributed to avian pox [2], and the virus may be contributing significantly to the continued decline of some populations. Two variants of the virus have been reported in native and non-native birds in the Hawaiian Islands. They differ in virulence, and have been shown to cause mortality among naïve Hawaìi `amakihi (*Hemignathus virens*), hereafter `amakihi, under experimental conditions [8]. One of these variants has been shown to have a very close phylogenetic relationship with canarypox, as have *Avipoxvirus* variants present in the Galapagos Islands [4]. In the Hawaiian Islands, co-infections of avian malaria (*Plasmodium relictum*) and *Avipoxvirus* in natural populations of forest birds is common and more frequent than expected by chance alone [9], [10]. Given the potential immunocompromising capabilities of pox viruses [11], [12], co-infection with *Plasmodium* may result in increased severity of acute malarial infections and recrudescence of chronic infections, with potential influences on both virulence and transmission of both pathogens.

The gold standard for diagnosing infection with *Avipoxvirus* continues to be both isolation of live virus and demonstration of the characteristic viral inclusion bodies (i.e. Bollinger Bodies) in fixed and stained sections of lesions that are typically collected at necropsy. Neither of these methods is effective in field studies of wild avian populations because the viral inclusion bodies typically occur in the dermis and are thus inaccessible to non-invasive sampling methods. While biopsy of these highly vascularized lesions under field conditions is possible, it is difficult in small passerines, where creation of an open wound may lead to subsequent secondary bacterial infections. As a result, most field studies of *Avipoxvirus* rely on presumptive diagnoses of lesions that are not able to differentiate viral infection from swellings that may be caused by knemidokoptic skin mites, bacterial infection, or mechanical injuries.

The relatively recent introduction of a highly efficient mosquito vector (*Culex quinquefasciatus*) and two avian pathogens (*P. relictum* and *Avipoxvirus*) to Hawaìi's isolated island ecosystem with naïve, highly susceptible avian hosts provides unique opportunities to investigate host-parasite-parasite co-evolution in a natural disease system. Development of an effective method for safely confirming and genotyping infection with *Avipoxvirus* in both avian hosts and arthropod vectors is critical for forming a better understanding of the population level impacts of this disease and how it interacts with other pathogens. Here, we describe a nested TaqMan Real-Time PCR method for the detection of *Avipoxvirus* in archived blood samples of Hawaiian birds with applications toward population-level analyses.

## Results

**Figure 1:**
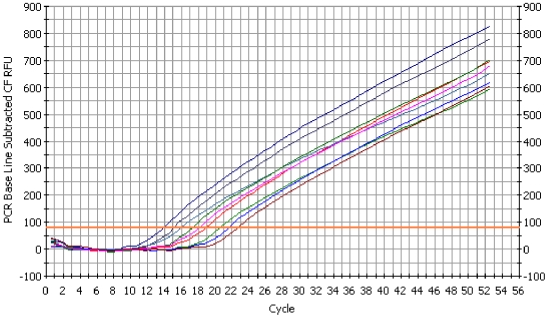
Real-time amplification of a serially diluted known positive sample. PCR base line subtracted curve fit data shows amplification of the *Avipoxvirus* 4b core protein gene from a 1∶2 serial dilution of first reaction PCR product using gDNA from *Avipoxvirus* culture lysate as template. The threshold for this reaction was 79.0 rfu.

The results from a Taqman Real-Time PCR completed on a serial dilution of first reaction products from a known positive sample (pox culture lysate from Variant 2, Hawaìi `iamakihi 15; [8]) are shown in Figure 1 and Table 1. In real-time diagnostic assays, a positive or negative result is often determined by the cycle number at which signal from a sample crosses a baseline threshold (Ct). Ct values for the serial dilution ranged from 13.9 to 22.7 with the differences between Ct values shown in Table 1. The dilution series shows Ct differences ranging from 0.7 to 1.6, which is close to the expected 1.0 cycle increase expected in a 1∶2 dilution series as the concentration of the target decreases [13].

**Table 1:** Ct values, Ct differences between dilutions, and final intensities for serial dilution of first reaction products from *Avipoxvirus* culture lysate.

Sample dilution	Ct Value (cycles)	Ct Difference (cycles)	Final RFU
2.0×10^−4^	13.9	--	825.81
1.0×10^−4^	15.2	1.3	781.28
5.0×10^−5^	15.9	0.7	694.47
2.5×10^−5^	17.3	1.4	652.49
1.25×10^−5^	18.1	0.8	594.54
6.25×10^−6^	18.9	0.8	702.34
3.12×10^−6^	20.5	1.6	616.03
1.56×10^−6^	21.7	1.2	681.56
7.81×10^−7^	22.7	1.0	606.69

We evaluated a total of 36 frozen packed blood cell samples and 22 corresponding plasma samples from wild Hawaiian honeycreepers. Seven of these samples represent known *Avipoxvirus* infections and were collected on the islands of Hawaìi and Molokài between 2002 and 2009 from three species of honeycreepers: `amakihi (n = 3), `apapane (*Himatione sanguinea*, n = 3), and ìiwi (*Vestiaria coccinea*, n = 1). Infections in these birds were confirmed either by successful culturing of *Avipoxvirus* or positive PCR screening of lesion samples taken at the time of blood sample collection. We evaluated plasma samples from four of these seven birds. In addition, we evaluated 29 frozen packed cell samples collected from wild `amakihi on the island of Hawaìi between 2002 and 2005 [10], [14]. All 29 of these birds had pox-like, smooth or scabby swellings on the feet or legs at the time of capture. Plasma samples collected from 18 of these 29 birds were also evaluated. Gel electrophoresis of products from the first reaction using packed cell samples (primers P1 [15] and PV4B.P5 [8]) revealed a band at approximately 450 bp in all but one individual, corresponding to the expected fragment size for *Avipoxvirus* product. Sequencing of a small number of these bands (n = 4) revealed that the bands do not represent the *Avipoxvirus* 4b core protein gene, but instead appear to originate from the avian host. The 450 bp band was not detected in any of the 22 plasma samples analyzed. Because these bands appeared in at least one first reaction for all known infected packed cell samples and 28 of 29 unknown packed cell samples, they served as an internal reference for the quality of each template DNA as well as the success of the first reaction. The one bird for which no first reaction band was observed in any of the three repeat reactions was considered to be a poor quality template and was removed from further consideration.

The nested TaqMan PCR amplified *Avipoxvirus* DNA from one out of seven packed cell samples from known pox-infected birds in three separate PCR reactions (Table 2). This sample (HAAM 26.1) produced a clear positive result in all reactions and was included as a positive control in reactions with unknown samples as well. The method presented here also amplified *Avipoxvirus* DNA from 13 of 28 packed cell samples of `amakihi with presumed but not confirmed pox infections (Table 3). In two of these 13 `amakihi, *Avipoxvirus* DNA was detected in all reactions. Three other `amakihi produced successful amplification of *Avipoxvirus* DNA in two of three reactions, and an additional three `amakihi were successful in one of two reactions. Successful amplification in only one of three reactions was observed in five `amakihi, while no successful amplification of *Avipoxvirus* DNA occurred in the remaining 15̀amakihi (1–3 successful first reactions per individual). *Avipoxvirus* DNA was not detected in plasma samples from four known infected samples and 18 samples with unconfirmed infections. Amplification was not detected for negative controls in any of the reactions, nor was any other sign of contamination observed. Bands from 11 of the PCR-positive birds (packed cell samples) were gel purified and sequenced, and all sequences were identified as the expected portion of the *Avipoxvirus* 4b core protein gene (Table 3). One bird was infected with variant 1, while eight individuals were infected with variant 2 [8]. Interestingly, two additional birds were infected with both variants based on the presence of numerous mixed peaks in the chromatograph at positions corresponding to expected single nucleotide differences between the variants.

**Table 2:** Ct values and final intensities from triplicate nested TaqMan Real-Time PCR reactions for packed cell samples from wild honeycreepers with confirmed *Avipoxvirus* infections.

Sample ID^1^	Ct	final RFU	Ct	final RFU	Ct	final RFU
HAAM 28.1 HI 2003	35.1	56.14	N/A	34.11	45.5	64.47
IIWI 3.1 HI 2003	45.8	29.87	49.6	49.45	N/A	41.32
HAAM 26.1 HI 2005	NR^3^	NR	**18.7**	**748.04**	**17.2**	**661.42**
APAP 14.1 MO 2003^2^	37.9	53.66	N/A	33.30	49.3	44.28
HAAM 15.4 HI 2003^2^	–	–	N/A	46.02	N/A	31.64
APAP 16.1 HI 2003^2^	–	–	N/A	25.10	N/A	42.52
APAP 30.1 HI 2009	–	–	N/A	29.23	46.1	53.55
lysate (+)	**14.2**	**1014.76**	**16.9**	**925.04**	**16.1**	**830.80**
dH2O (−)	32.8	204.22	45.6	74.06	49.5	46.07
dH2O (−)	30.3	90.75	45.6	75.01	49.7	45.19

Successful amplifications (Ct<25 and final RFU>425) are indicated in **bold**. All values are based on PCR Base Line Subtracted Curve Fit Data as calculated using iCycler version 3.1 software (BioRad). The threshold intensity was 23.4 rfu for the first reaction, 51.4 rfu for the second reaction, and 41.7 rfu for the third reaction.

1 Samples are identified by species, island and year of capture. Abbreviations are as follows: HAAM, Hawaìi `amakihi (*Hemignathus virens*); IIWI, ìiwi (*Vestiaria coccinea*); APAP, `apapane (*Himatione sanguinea*); HI, Hawaìi; MO, Molokài.

2 Laboratory *Avipoxvirus* isolates cultured from these individuals are included in Jarvi, et al., 2008 [8].

3 NR indicates the sample was not included in that reaction, a dash (−) indicates a failed first reaction and potential false negative, N/A indicates that no Ct value was assigned because the signal for the sample never reached the threshold intensity for that reaction.

**Table 3:** Ct values, final intensities and sequencing results from triplicate nested TaqMan Real-Time PCR reactions for packed cell samples from wild `amakihi with presumptive pox lesions.

	Ct	final RFU	Ct	final RFU	Ct	final RFU	*Avipoxvirus* variant^2^
6779	N/A	21.77	N/A	47.9	49.4	13.12	
7216	–1	–	50.8	54.99	49.9	14.63	
7379	**18.1**	**647.43**	**18.2**	**764.55**	**14.8**	**624.27**	2
7396	N/A	−8.85	–	–	49.5	13.83	
7596	–	–	44.9	61.56	34.2	115.39	
8495	25.7	98.83	N/A	24.54	49	14.88	
10232	–	–	–	–	50.1	8.68	
10291	**23.8**	**441.35**	36.7	396.51	1.7	9.22	2
10302	**18.9**	**762.18**	45.8	57.83	33.5	48.41	2
10332	**23**	**628.95**	N/A	36.45	45.2	22.2	NS
10342	–	–	–	–	37.5	276.06	
10588	**20.4**	**584.71**	38.3	76.88	44.4	29.04	2
10630	N/A	−2.08	N/A	42.87	34.7	38.88	
10643	**17.4**	**751.9**	–	–	42.7	45.71	2
10652	N/A	10.11	N/A	4.49	49.5	14.46	
10716	**19.1**	**696.3**	43.6	66.56	–	–	2
11350	43.1	46.76	N/A	36.22	45.8	20.84	
11408	–	–	**24.3**	**692.91**	**15.4**	**596.31**	2
11458	**22.4**	**707.45**	39.2	75.03	**16.3**	**486.49**	2
12657	**23.1**	**430.5**	**22.3**	**742.46**	28.5	40.31	NS
12717	**22**	**647.68**	39.8	74.78	**20.6**	**497.12**	1,2
12818	N/A	24.08	48.7	54.75	45.2	25.04	
12847	49.8	40.76	42.8	74.77	46	19	
12858	N/A	−4.86	45.8	61.09	**17.1**	**567.41**	NS
13916	–	–	**21.9**	**557.07**	N/A	−1.13	1
16562	N/A	8.18	45.3	59.42	49.5	9.48	
16922	42	52.16	–	–	45.8	16.7	
17331	N/A	25.82	40	69.97	48.3	23.75	
HAAM 26.1	**19.7**	**815.81**	**17.9**	**854.29**	**14**	**667.18**	1,2
lysate (+)	**18.2**	**731.57**	**16.3**	**825.46**	**13.9**	**602.86**	2
dH2O (−)	N/A	3.98	N/A	37.13	49.6	20.37	
dH2O (−)	N/A	1.02	40.7	78.88	49.9	11.17	

Successful amplifications (Ct<25 and final RFU>425) are indicated in **bold**. All values are based on PCR Base Line Subtracted Curve Fit Data as calculated using iCycler version 3.1 software (BioRad). The threshold intensity was 37.0 rfu for the first reaction, 48.3 rfu for the second reaction, and 10.8 rfu for the third reaction.

1 A dash (−) indicates a failed first reaction and potential false negative, N/A indicates that no Ct value was assigned because the signal for the sample never reached the threshold intensity for that reaction.

2 Variant numbers correspond to *Avipoxvirus* clusters 1 and 2 as previously described [8]; NS indicates not sequenced.

## Discussion

We present here a successful method for amplifying the 4b core protein gene of *Avipoxvirus* from archived field blood samples. This method detected *Avipoxvirus* DNA in one of seven wild honeycreepers with confirmed *Avipoxvirus* infections and in 13 of 28 wild `amakihi with pox-like lesions. The rate of detection of *Avipoxvirus* DNA in blood samples from birds with confirmed infection was disappointingly low. This procedure is therefore not a useful diagnostic when applied alone; however, it can provide confirmation of a presumptive diagnosis based on the presence of pox-like lesions when amplification is successful. Perhaps more importantly, this nested TaqMan Real-Time PCR provides a method for genetic characterization and confirmation of suspected pox infections that would not otherwise be available from archived field samples.

Prior to the development and application of this technique, it was unknown whether such archived samples could be used for the study of *Avipoxvirus* in wild populations of birds. Relatively little is known about the life cycle of *Avipoxvirus* in hosts other than domestic poultry, and it is possible that the virus may not always be present in the blood of apparently infected birds or may be present at titers below the detection limit of this method. Fowlpox, one of 10 currently recognized species of *Avipoxvirus*, has been detected by live virus isolation in the buffy coat portion of the blood of intravenously infected chickens [16], as well as by real-time PCR in the buffy coat of one of nine chickens infected via wing web inoculation [17]. However, most PCR-based studies of fowlpox virus and other *Avipoxvirus* species continue to rely on lesion scrapings or biopsies as a source of template DNA [4], [18]. To our knowledge, this is the first protocol to successfully amplify *Avipoxvirus* from the blood of naturally infected, wild passerines. Real-time methods have had mixed success in detecting other orthopoxvirus species in blood, with one group reporting successful detection of mouse pox virus in the spleen and lung but not blood of experimentally infected mice [19] and another group reporting relatively high rates of detection of monkeypox virus DNA in the blood of experimentally infected monkeys [20]. Other studies have demonstrated very low rates of detection of vaccinia virus DNA in the blood of individuals recently vaccinated for smallpox [21], [22]. One of the few studies applying real-time diagnostics to blood samples from natural infections reported successful amplification of bovine vaccinia from a single human blood sample [23]. Most successful amplification of pox virus DNA from blood samples of experimentally infected animals or vaccinated humans are within 11–21 days post infection [20], [21], suggesting that the presence of viral DNA in the host bloodstream may be of limited duration. Our lack of detection in plasma samples and low rate of detection in packed cell samples from known infections agree with these studies and is most likely a result of the lack of viremia at various time points during infection.

From the 28 Hawaìi `amakihi with a presumptive diagnosis of avian pox included in this study, we were able to amplify pox in at least one of three reactions from 13 birds. This detection rate is slightly higher than that observed in `amakihi with confirmed *Avipoxvirus* infections (1/3) and much higher than observed in known infected `apapane (0/3) and ìiwi (0/1). While sample sizes are too low to draw any firm conclusions, further investigation of species-specific differences in detectability of *Avipoxvirus* infection that may reflect both magnitude and duration of viremia in different avian hosts appear warranted.

Of the 13 `amakihi from which *Avipoxvirus* DNA was successfully amplified, eight individuals were positive in only one reaction. While this could be explained as contamination, methods for controlling contamination were rigorously followed and no signs of contamination were observed in negative control samples. Furthermore, levels of target DNA close to the detection limit of a method have been shown to produce inconsistent real-time results [24], [25]. We therefore conclude that a lack of amplification in repeat reactions for these samples is most likely due to low titer viremia in the blood of these individuals. Future applications of this method may be improved by the use of viral-enriching extraction techniques [26].

Failure to amplify pox DNA from the remaining 15 of 28 wild `amakihi could be because the virus is not present in the blood of these birds or may be present in very low titers. Alternatively, the initial presumptive diagnosis of *Avipoxvirus* infection based only on the presence of tumor-like lesions or swelling may have been incorrect. Pox-like lesions or scars can be caused by injury, infestation with knemidokoptic skin mites, and/or secondary bacterial infections, and are not always confirmed by more stringent methods [2].

In a previous study of cultured *Avipoxvirus* isolates from Hawaìi, 4b core protein gene sequences sorted equally into one of two clusters that were designated variant 1 and variant 2 [8]. In contrast, the majority of the wild infections confirmed in the current study were identified as variant 2. Experimental studies indicate that variant 2 is more virulent in `amakihi, producing larger, more proliferative lesions than variant 1 [8]. Interestingly, a loss of virulence has been linked to lower viremia in sheeppox virus [27]. Therefore, it is possible that the higher rate of detection of variant 2 in this study is due to the production of higher viremias by the more virulent variant.

The detection of two individuals infected with a mix of *Avipoxvirus* variants 1 and 2 was unexpected given the lack of detection of genetically mixed infections in the limited number of lesion samples in a previous study [8]. This may be an indication that simultaneous *Avipoxvirus* infections are more readily detected in blood samples than from lesion biopsies, particularly if viral genotypes are segregated by lesion and missed during biopsy. This has important implications for how lesions are sampled for diagnosis and genotyping and for our understanding of their pathogenesis. Our detection of mixed genotype *Avipoxvirus* infections in two `amakihi also suggests that simultaneous infections may be more common than anticipated and highlights an additional source of complexity in the transmission of these diseases in Hawaìi. Population level studies will be required to determine the distribution of each variant and the extent of co-infection between pox variants and between *Avipoxvirus* and avian malaria as well. To that end, this new non-invasive method to confirm and genotype *Avipoxvirus* infections should prove a useful and highly informative tool. Because of the close relationship of Hawaiian *Avipoxvirus* and Galapagos *Avipoxvirus* to canarypox [4], [8], we anticipate that the primers, probe and method described here will also be applicable to other avian populations with only minor modifications and thus may be a useful tool in global studies of the epidemiology of *Avipoxvirus*.

## Materials and Methods

### Sample Collection

Collection of field samples was approved by the University of Hawaìi Institutional Animal Care and Use Committee, protocol 00–035. Blood samples were collected by jugular venipuncture with heparinized 26 gauge insulin syringes from 29 wild `amakihi with smooth or scabby swellings that superficially resembled pox lesions. These blood samples were obtained on the Eastern slope of Mauna Loa and Kilauea Volcanoes on the island of Hawaìi as part of a larger study of the transmission of avian malaria and pox virus in native and non-native forest birds (NSF Biocomplexity of Introduced Avian Diseases in Hawaìi; [14]). Birds were mist-netted, banded, bled and released. Immediately after blood was drawn, it was transferred to heparinized microhematocrit tubes and centrifuged with a battery-operated field centrifuge to separate plasma from cells. The microhematocrit tube was scored with a file and broken just above the boundary of the buffy coat and plasma, and plasma was transferred to an empty, sterile 0.5 ml vial before freezing. Packed lymphocytes and erythrocytes were removed with a filter-tipped pipetter, transferred to an equal volume of lysis buffer (2% sodium dodecyl sulfate, 0.1 M EDTA, 0.1 M Tris, pH 8.0) in a sterile 0.5 ml vial and frozen. These samples are referred to as “packed cells” throughout and contain packed red blood cells and a portion of the buffy coat.

Blood samples and tissue samples from pox-like lesions were also obtained from an additional three wild `amakihi, one wild ìiwi, and three wild `apapane. *Avipoxvirus* infection in six of these birds was confirmed by live virus isolation and propagation as previously described [8]. *Avipoxvirus* infection in the seventh bird (APAP 30.1) was confirmed using the real-time method described here.

### Samples and DNA extraction

DNA was extracted from pox culture lysate as previously described (Variant 2, Hawaìi `amakihi 15; [8]) for use as a positive control in all reactions. DNA was extracted from packed cells and a single lesion tissue sample using the Qiagen DNeasy Animal Tissue Kit following manufacturer's protocols. Blood samples were stored in lysis buffer at −80°C for up to three years prior to DNA extraction; genomic DNA was stored at −80°C for as many as eight additional years prior to real-time PCR analysis. “Blank” samples containing no tissues were included in extractions and carried through subsequent steps to monitor for contamination.

### PCR Amplification of *Avipoxvirus* DNA

A nested PCR approach was used to amplify a portion of the *Avipoxvirus* 4b core protein gene. In the first reaction, 1 µl of genomic DNA from field samples or pox culture lysate, or 1 µl of unextracted plasma, was used as template in 25 µl reactions containing 1X PCR buffer, 1.5 mM MgCl_2_, 200 µM each dNTP, 0.4 µM each of primers P1 [15] and PV4B.P5 [8], and 1.25 units GoTaq Flexi (Promega, Madison, WI, USA). Reactions were subjected to an initial denaturing period of 2 minutes at 96°C, followed by 55 cycles of 96°C for 1 minute, 52°C for 1 minute, and 72°C for 1 minute, with a final extension step of 7 minutes at 72°C. Products from the initial PCR were diluted 1∶80,000 in water and 1 µl of dilution was used as template in a TaqMan real-time PCR assay. Primer 1F (5′-TCC TTG TAA AAG CGA TAC AGG AA-3′) and primer 1R (5′-CCC CTT AAC ATG TGC TAA CAA-3′) produce a 234 bp fragment within which lies the Pox1 probe (5′-/56-FAM/CAG CGT GAT GAA GAC GCT AA/3BHQ_1/-3′), which is dual labeled with a 5′-56-FAM reporter and a 3′-Black Hole Quencher (Integrated DNA Technologies, Coralville, IA, USA). The real-time PCR assays were run as 50 µl reactions containing 0.4 µM primer 1F, 0.4 µM primer 1R, 0.4 µM Pox1 probe and 25 µl iQ Supermix (BioRad, Hercules, CA, USA). Reactions were run on a BioRad iCycler thermal cycler equipped with an iQ Multi-Color Real-Time PCR detection system (BioRad) using a 3 step PCR method consisting of an initial denaturation step at 95°C for 3 minutes, followed by 55 cycles of 95°C for 30 seconds, 55°C for 15 seconds, and 63.3°C for 30 seconds. Reactions for packed cell samples were repeated in three separate reactions, with samples run in a different random order each time; reactions for plasma samples were run once. Precautions to reduce and prevent contamination were taken during set-up of all reactions and included the use of a CloneZone PCR workstation (USA Scientific, Ocala, FL, USA) that was cleaned with sodium hypochlorite solution and subjected to a minimum of 15 minutes UV irradiation between reactions. Negative controls (water instead of template DNA) were included in all reactions, and all reactions were run in individual 0.2 ml tubes with attached optical dome cap (BioRad). Data was analyzed using the PCR Base Line Subtracted Curve Fit function of iCycler version 3.1 software (BioRad). Successful amplification of the target *Avipoxvirus* 4b core protein gene was determined based on Ct values below 25 and final signal intensities above 425 rfu. In addition to collecting real-time information during the run, products of both PCRs were analyzed by electrophoresis on 1.5% agarose gels (SeaKem, Lonza, Switzerland) stained with ethidium bromide. In order to evaluate the real-time assay and to determine the best dilution value between the first and second reaction, a 1∶2 serial dilution (2.0×10^−4^ to 7.81×10^−7^) of PCR product from the first reaction positive control (pox culture lysate) was prepared and run using the real-time conditions described above.

### Sequencing of PCR Products

To confirm the amplification of *Avipoxvirus* DNA in the real-time assay and to determine the identity of observed bands in the initial reaction, PCR products were purified via gel excision. Products were run on a 2% low-melt agarose gel (Fisher Scientific, Pittsburgh, PA, USA) and bands of interest were excised and purified following the manufacturer's protocol using the QIAquick Gel Extraction Kit (Qiagen, Valencia, CA, USA) with the final elution in 30 µl Buffer EB. Purified PCR products were direct sequenced in both directions using the appropriate PCR primers (ASGPB, University of Hawaìi at Manoa). Resulting sequences were hand corrected and aligned using Sequencher v. 4.2 (GeneCodes Corporation, Ann Arbor, MI, USA) and identified via BLAST search on GenBank. All sequences are available in GenBank under accession numbers GU982265 – GU982280.
